# A Malicious Pattern Detection Engine for Embedded Security Systems in the Internet of Things

**DOI:** 10.3390/s141224188

**Published:** 2014-12-16

**Authors:** Doohwan Oh, Deokho Kim, Won Woo Ro

**Affiliations:** School of Electrical and Electronic Engineering, Yonsei University, 50 Yonsei-ro, Seodaemun-gu, Seoul 120-749, Korea; E-Mails: ohdooh@yonsei.ac.kr (D.O.); nautes87@yonsei.ac.kr (D.K.)

**Keywords:** intrusion detection system, Internet of Things, pattern detection, Wu-Manber

## Abstract

With the emergence of the Internet of Things (IoT), a large number of physical objects in daily life have been aggressively connected to the Internet. As the number of objects connected to networks increases, the security systems face a critical challenge due to the global connectivity and accessibility of the IoT. However, it is difficult to adapt traditional security systems to the objects in the IoT, because of their limited computing power and memory size. In light of this, we present a lightweight security system that uses a novel malicious pattern-matching engine. We limit the memory usage of the proposed system in order to make it work on resource-constrained devices. To mitigate performance degradation due to limitations of computation power and memory, we propose two novel techniques, auxiliary shifting and early decision. Through both techniques, we can efficiently reduce the number of matching operations on resource-constrained systems. Experiments and performance analyses show that our proposed system achieves a maximum speedup of 2.14 with an IoT object and provides scalable performance for a large number of patterns.

## 1. Introduction

The Internet of Things (IoT) is an emerging paradigm shift in the use of the web from communicating with end-user devices to connecting physical objects by themselves [1–3]. In this paradigm, many objects of daily use around us will be embedded with smart sensors and computational resources and will be connected to networks in one form or another. Wireless sensor network technologies and modern embedded computing systems will be developed to meet this new emerging paradigm.

A wireless sensor network (WSN) is a network of sensor nodes that detect and record environmental data and send them to a sink node. The sink node further processes received data and communicates with outer nodes. The sensor nodes are resource-constrained devices with limited storage and processing capabilities. The Internet Protocol (IP) is a heavyweight protocol that is considered inadequate for the sensor nodes. Therefore, a conventional WSN is a non-IP network, where each sensor node only communicates with neighboring nodes or the sink node. However, with the emergence of IoT, the use of IP in resource-constrained WSNs has been requested. In light of this demand, IP version 6 (IPv6) over low-power personal area networks (6LoWPANs) has been standardized [4]. With the advance of 6LoWPAN, it becomes possible to use IP in a resource-constrained sensor node of WSNs. As a result, each sensor node can be accessed anywhere at anytime by authorized devices using 6LoWPAN.

As an ever-increasing number of emerging WSNs use IP, the number of smart objects connected to the Internet increases. More than 10 billion smart objects will be operated and connected through the Internet together with using various applications, such as e-health, gas and electricity meters, *etc*. The networking company, Cisco, has announced that more than 50 billion devices are expected to be connected to the Internet, producing terabytes of data per second, by 2020 [5]. As devices connected to the Internet become increasingly pervasive, security becomes an increasingly critical issue [6]. Indeed, the IoT devices seamlessly gather personal data using embedded sensors. Although only authorized users can access these devices, there is always a certain level of threat that the security system can become vulnerable to illegal activities and attacks, as in desktop computers and server systems. Therefore, an effective security system is needed to protect smart objects against such attacks.

An intrusion detection system (IDS) monitors networks for malicious attacks on inbound and outbound packets by using a misuse detection scheme [7,8]. This scheme uses a pattern-matching algorithm to check predefined pattern sets consisting of intrusion signatures [9]. Each of these signatures is defined in the context of payloads that have previously been revealed as malicious attacks. The IDS inspects the context of the payloads by checking them against the predefined signature set. Therefore, the pattern-matching engine is one of the most important features in network security applications designed to search for malicious patterns. However, it is difficult to operate a conventional pattern-matching engine on smart objects, because most of them are resource constrained in terms of power, processing and memory space [10–12].

In this paper, we propose a new malicious pattern-detecting system that has low computational complexity and requires a small amount of memory in order to protect IoT objects against breaches of security. The proposed system is developed based on a traditional pattern-detection algorithm that is widely used for computer security applications. We have found out that some of the target data can be skipped without inspecting operations more closely than they are in the traditional security algorithm. The amount of data that can be skipped is precomputed in our proposed system as auxiliary shift values. Furthermore, we limit the memory usage of the traditional algorithm to render it suitable for resource-constrained smart objects. In order to prevent performance degradation due to this limitation, our proposed algorithm reduces the required number of additional matching operations through early decision on character matching operations.

We make the following three contributions in this paper to improve the performance of malicious pattern-matching systems operating on smart objects:

- With the auxiliary shift value, a large amount of data that are not matched on any patterns can be skipped.
- Within patterns that have identical prefix values, the information obtained by character matching can be used to determine the early termination of the matching operation.
- Our proposed algorithm reduces memory usage for malicious pattern-detecting processes and, therefore, enables the incorporation of resource-constrained smart objects into the security system.

To test the performance of the proposed algorithm, we performed experiments using a smart object embedded with an image sensor and a computing resource. Our algorithm attained a performance gain of 10% over the traditional algorithm by applying auxiliary shifting. Moreover, the early decision scheme provided an additional speed-up of 1.87. Finally, the proposed algorithm provided a maximum speed-up of 2.14 compared with the traditional algorithm.

The rest of the paper is organized as follows. In Section 2, the details of the traditional pattern-matching algorithm and its inefficiency are presented and related research in the area is surveyed. In Section 3, we detail our proposed algorithm. Analyses of computational complexity for the proposed algorithm are presented in Section 4. Experimental results and analyses are presented in Section 5. This paper is concluded in Section 6.

## 2. Background Knowledge and Related Work

### 2.1. Security in the Internet of Things

In the IoT paradigm, physical devices can be connected to the Internet and allowed to utilize a massive amount of data information. Each device can send and receive necessary data and can make its own decision based on the information. For example, in healthcare applications, the IoT technology can help to cope with emergency situations with rapid responses [3]. Patients will wear medical sensors to monitor their physiological statistics, such as body temperature, blood pressure and breathing activity. The data collected from the sensors will be integrated into the global healthcare applications. These applications continuously gather and look over the data in order to monitor the patients. If any kind of unusual conditions are detected, the system alerts the relevant medical center, as well as the patient.

Security is one of the most important features to be taken into account in designing the IoT, because of the connectivity and sensitivity of the data collected [6,10,13]. Although the IoT networks can only be accessed by authorized users, several kinds of attacks can be mounted against networks. These attacks are aimed at disrupting network communication or collecting private data. For instance, the denial-of-service (DoS) attacks that occur in the network layer rapidly disrupt communication. The attacker repeatedly sends packets to the target device and, thus, exhausts its computational resources. An eavesdropping attack is another serious security threat. A malicious node actively steals messages transmitted through the networks and then joins the network as a legitimate node by using the stolen messages. Furthermore, the node carries out attacks against Internet hosts. An application layer attack causes errors in the operating system of a device and is able to bypass authorized access controls.

Intrusion detection systems for the IoT networks are required to detect malicious activities in the networks. However, smart objects connected to the IoT networks have lower computing power than the general computing systems, even though they are more powerful than the traditional sensor node previously used in WSNs [14]. It is difficult to adapt the previously developed security systems for desktop computers to the smart objects due to the hardware restrictions on computing power, memory size and battery life.

### 2.2. Traditional Malicious Pattern Detection

**Figure 1.**
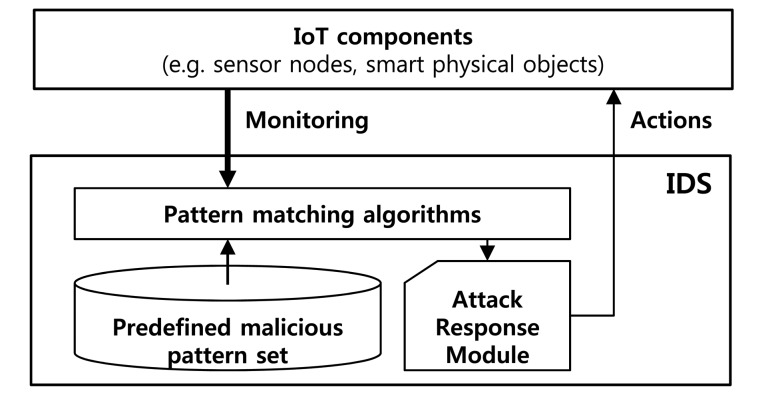
Architecture of intrusion detection system.

Most intrusion detection systems detect malicious attacks by using pattern-matching algorithms with predefined malicious attack patterns, as shown in Figure 1. A multiple string-matching algorithm has been proposed to find all patterns of a finite pattern set *P* = {*p*^1^, *p*^2^, …, *p^n^*}, in a text *T* = {*t*_0_*t*_1_ … *t_l_*_−1_} of length *l*. The patterns and text are sequences of characters from an alphabet Σ. The pattern set is defined according to empirically-determined malicious patterns (malicious signatures). The text is constituted by inbound and outbound packets from networks or system files inside target devices.

The Wu–Manber (WM) algorithm [15] is one of the fastest multiple pattern-matching algorithms and is widely used as a malicious pattern-detection engine. It is composed of two stages. The first is a preprocessing stage to construct three required tables, the shift, hash, and prefix tables. The second stage is a pattern-matching stage to perform matching operations using these tables. The second stage accounts for most of the execution time.

The shift table provides the magnitude of the shift distance for each block. Therefore, the table has Σ*^B^* entries to present all possible combinations of characters in the block, where Σ is the number of characters used in the pattern set and *B* is the size of the block. Each entry of the shift table indicates the maximum distance to the next possible matching block.

The maximum shift distances of the entries can be calculated by a heuristic method using the following two cases:

Case 1: When the block is not related to a pattern's substring consisting of the first *m* characters of the pattern, the entry has a maximum value of *m* − *B* + 1 (*m* ≥ *B*), where *m* is the minimum pattern length.
Case 2: When the block is related to a substring of the patterns, the entry has the smallest value of *m* − *q*, where *q* is the position with which the block is related in a pattern for *B* ≤ *q* ≤ *m*.

The hash table contains all entries of the shift table with zero shift value. Each entry in the hash table points to the starting address of patterns that have an identical suffix value. The prefix table stores prefix values of the patterns and provides the prefix values before initiating the character matching operation.

**Figure 2.**
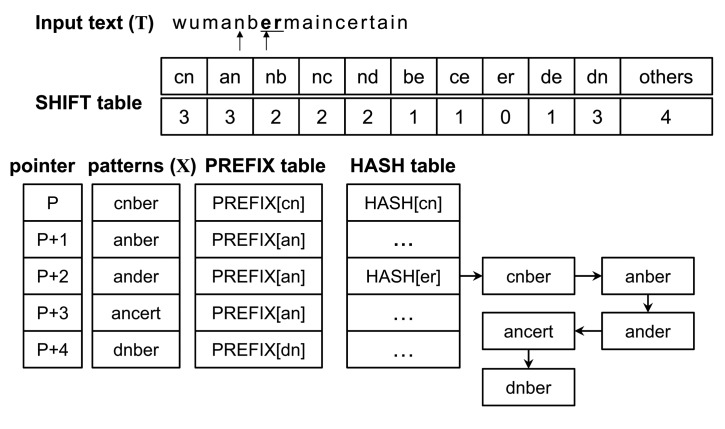
Wu–Manber multiple pattern-matching algorithm.

The matching operation is represented through the example shown in Figure 2, and the original Wu–Manber algorithm is described as follows:

Step 1: Compute the hash value *h* from the current block and check the shift value of the index *h* in the shift table. If this shift value is zero, go to the next step. Otherwise, the block moves to the right by as many characters as the shift value. Repeat Step 1.
Step 2: Compute the hash value *p* from the prefix characters of the current block. Match *p* with the prefix of a pattern listed in the *h* index in the HASH table. If matched, go to the next step. Otherwise, traverse to the next pattern in the list until all patterns have been checked and then return to Step 1 after shifting the block by one character.
Step 3: The remaining characters of the pattern, the prefix for which has been matched with *p*, are compared with the characters of the input text. The character matching operation continues until a mismatch occurs. When a mismatch is found, return to Step 2. When all characters in the pattern match those of the input text, the pattern is found. Return to Step 2.

In the figure, *h* and *p* are represented as *nb* and *um*, respectively (assuming that the size of the block and that of the prefix is two characters). Since the index *nb* in the shift table has the shift value two, the procedure goes to Step 1 after moving the scan window by two characters. Following this, *h* and *p* become *er* and *an*, respectively, and the shift value of the index *er* is zero. Thus, the procedure advances to Step 2. The five prefixes of the patterns listed in the *er* index in the hash table are subsequently evaluated using *an*, whereas *anber*, *ander* and *ancert* should perform character matching operations on the input text according to Step 3. Because all characters in *anber* match with the input text, the pattern is found. The block hence moves forward by one character.

Observation 1: The algorithm always moves to the right by a single character after completing Step 2. However, there is the possibility of shifting more than one character using a bad-character shift. The current block cannot be matched to the suffix value of any other pattern following the completion of Step 2, because the algorithm has satisfactorily examined the block in Steps 2 and 3. Therefore, the current block can be considered a non-suffix value.

Observation 2: The algorithm is inefficient in that all prefix values in Step 2 and all patterns in Step 3 need to be looked up. Since patterns have been sorted based on their suffix values in the original algorithm, the prefix values need to be compared for all patterns listed in the *er* index in the hash table. Moreover, all patterns with the prefix *an* need to be compared to the input data stream in Step 3.

Observation 3: The block size can be expanded to reduce the number of patterns per HASH table entry. However, this approach drastically increases the size of the hash table in proportion to a power of Σ. That is, the hash table requires more than 4 GB of memory when *B* = 4 and Σ = 256. Furthermore, the prefix table has an analogous problem to that faced by the hash table in this context.

### 2.3. Related Work

The Boyer-Moore algorithm is a well-known single-string matching algorithm [16], and numerous single-string matching algorithms have been developed based on it [17–19]. The algorithm compares characters in the text from the rightmost character in a pattern. When a mismatch occurs, the algorithm determines the maximum distance to shift the scan window [16]. The algorithm has *O*(*mn*) execution time in the worst case, where *m* is the length of the pattern and *n* is the length of the text string [20].

A multiple pattern-matching algorithm based on the Boyer-l-Moore algorithm, the WM algorithm expands it by using an effective bad-character shift method [15]. The algorithm uses the concept of a scan window, which is a block consisting of *B* characters, where the value of *B* is commonly two or three [15]. The algorithm hashes the characters in the scan window and uses the hash table to solve collisions. The algorithm is highly efficient and has been embedded in the *agrep* command, which is available on the UNIX operating system [21].

Commentz and Walter [22] integrated the Aho–Corasick multiple pattern-matching approach [23] into the Boyer–Moore algorithm. Lecroq [24] introduced a fast single-string matching algorithm based on the Wu–Manber algorithm. Oh and Ro proposed pattern rearrangement methods for the Wu–Manber algorithm and accelerated the matching procedure by exploiting parallelism on multicore processors [25,26]. Hong *et al.* [27] proposed an improved Wu–Manber algorithm using the quick search algorithm [19]. They employed the head table to determine whether the characters in the scan window are a prefix of the patterns. Dai [20] also suggested an algorithm based on the Wu–Manber algorithm and the quick search algorithm, which considers an additional character next to the scan window and applies the quick search algorithm. Dai also analyzed all cases due to the additional character to maximize shift distance. Lin *et al.* [28] proposed a hybrid approach based on the Aho-Corasick algorithm and the WM algorithm. They also developed a backward hashing (BH) algorithm by modifying the WM algorithm to lengthen the average skip distance of the shift table.

Zhou *et al.* [29] proposed a variant of the Wu–Manber algorithm to cope with large-scale pattern sets. They used multi-phase hash and dynamic-cut heuristics to reduce memory usage and to choose an optimal size of the scan window. They efficiently improved the performance and reduced the required memory size for a large scale of patterns. However, the Wu–Manber algorithm still performed better than their method in their experiments when the number of patterns was fewer than 30,000 [20]. Liu *et al.* [30] also emphasized performance degradation with large-scale patterns and proposed their decomposition according to length. Although their method improved the performance by partitioning large-scale patterns, the matching operations of the algorithm are identical to the original Wu–Manber algorithm and, hence, inherit its inefficiency.

Zhang *et al.* [31] suggested an address-filtering method based on the Wu–Manber algorithm. The algorithm attempts to reduce the number of unnecessary matching operations by scanning a linked list within the prefix table [15]. However, their proposed approach sorts the prefix table according to address values, and there still exist inefficient address comparison operations on the patterns, each of which has a different suffix. Moreover, the algorithm cannot reduce the number of matching operations when the prefix table has a large number of patterns with an identical prefix within a suffix value.

## 3. Reducing Unnecessary Matching Operations on Embedded Systems

In this section, we introduce an advanced algorithm to skip and eliminate unnecessary matching operations in order to mitigate performance degradation caused by limitations on memory usage in the context of IoT. We first introduce the additional shifting method following prefix matching operations, followed by a technique to reduce matching operations and efficient ways to reduce the required number of comparisons.

### 3.1. Auxiliary Shift Value

As stated in Step 2 of the WM algorithm, the scan window always moves to the right by one character after all patterns listed in the *h* index in the hash table have been evaluated. However, we can move the window to the right by more than one character, and this can benefit processing speed. As mentioned in Observation 1, there is no possibility of finding patterns with the current block as their suffix value following Step 2. Therefore, we can recalculate a shift value corresponding to the current block by excluding these patterns. This shift value must be equal to or greater than one. This means that the scan window can move by more than one character following Step 2.

#### Definition 1

*The auxiliary shift value (AS, for short) is the minimum among the shift distances of an entry calculated from P, excluding patterns having the relevant entry as their suffix*.

The preprocessing steps of the shift table, including AS, are as follows: SHIFT[*h*] indicates the shift value of the *h* index and ASHIFT[*h*] marks an AS for the *h* index.

(1) The shift table has Σ*^B^* entries presenting all possible combination of characters in the block. All entries have one integer value initialized as *m* − *B* + 1.
(2) Compute all hash values *h*s for all substrings of length *B* from *p*′*^l^* = {*a*_0_*a*_1_…*a_m_*} for 0 ≤ *l* ≤ *n, B* = {*a_j_*_−_*_B_*_+1_…*a_j_*} for *B* ≤ *j* ≤ *m*, where *a* is from Σ. Set SHIFT[*h*] to min{m − *j* + 1, SHIFT[*h*]}. If SHIFT[*h*] = 0, the entry related to *h* has one more integer value named ASHIFT[*h*]; set ASHIFT[*h*] as the previous SHIFT[*h*]. At this time, if ASHIFT[*h*] *≠ NULL*, set ASHIFT[*h*] to min{previous SHIFT[*h*], ASHIFT[*h*]}.

**Figure 3.**
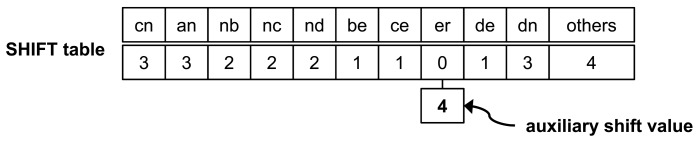
Shift table with auxiliary shift values.

The shift table with AS for a pattern set *X* in Figure 2 is shown in Figure 3. The only entry having zero shift value is *er;* SHIFT[*er*] = 0. Therefore, the *er* entry has an AS. This AS has the least shift value among the values of SHIFT[*er*], except for a zero value. For this pattern set, the AS is four.

Because we have added auxiliary shift values to the shift table, the matching operation of Step 2 in WM is modified as follows:

Step 2: Compute the hash value *p* from the prefix characters of the current block. Match *p* with the prefix of a pattern listed in the *h* index in the hash table. If matched, go to the next step. Otherwise, traverse to the next pattern in the list until all patterns have been checked. When all patterns have been checked, shift the block as provided in ASHIFT[*h*] and return to Step 1.

**Figure 4.**
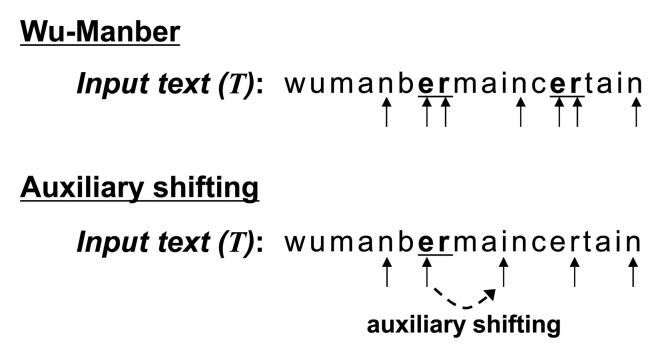
Examples of matching procedures for Wu–Manber (WM) and auxiliary shifting.

We compare matching procedures in WM and WM with auxiliary shifting in Figure 4. The character sequence of the initial block is “*nb*” in the input text (*T*). Both Wu–Manber and auxiliary shifting move the block to the right by two characters, since SHIFT[*nb*] = 2 in the shift table, as shown in Figure 3. The block then indicates the sequence “*er*” for which SHIFT[*er*] = 0. In this case, following the completion of Step 2 for the entry “*er*,” Wu–Manber shifts by only one character, and the next block has the sequence “*rm.*” On the other hand, auxiliary shifting shifts the block right by ASHIFT[*er*] = 4; the next block is “*in.*” Therefore, auxiliary shifting can skip three characters more than Wu–Manber.

As a result, using auxiliary shifting enables one to skip more characters in an input text than WM. Therefore, the number of hashing operations required in Step 1 decreases, as does the overall execution time for the matching procedure.

### 3.2. Early Decision Method

There are two cases where we can reduce unnecessary matching operations in Steps 2 and 3 of the Wu–Manber algorithm if we sort patterns with identical suffixes according to the prefix values as follows:

- In the prefix matching in Step 2, sorted patterns in the hash table provide necessary information to look up the prefix table to decide whether the remaining prefixes need to be compared.
- In the character matching in Step 3, although several patterns have an identical suffix value and an identical prefix value, a comparison of the characters can provide necessary information regarding whether the remaining sorted patterns need to be compared.

In our proposed algorithm, the patterns in each entry of the hash table are sorted in ascending alphabetical order. Using this sorted pattern set, we have modified Steps 2 and 3 of the WM with auxiliary shifting, and they are given as follows:

Step 2: Compute the hash value *p* from the prefix characters of the current block. Match *p* with the prefix of a pattern listed in the *h* index in the hash table. If matched, go to the next step. Otherwise, if the prefix does not match and is greater than *p* or if all of the patterns have been checked, shift the block as given in ASHIFT[*h*] and return to Step 1. If the prefix is smaller than *p*, traverse to the next pattern in the list.
Step 3: The rest of the characters of the pattern, the prefix of which is matched with *p*, are compared to the characters of the input text. The character matching operation continues until a mismatch occurs. When a mismatch is found and the character value of the pattern is greater than that of the input text, shift the block as in ASHIFT[*h*] and return to Step 1. Otherwise, all characters in the pattern match those of the input text, and the pattern is found. Return to Step 2.

**Figure 5.**
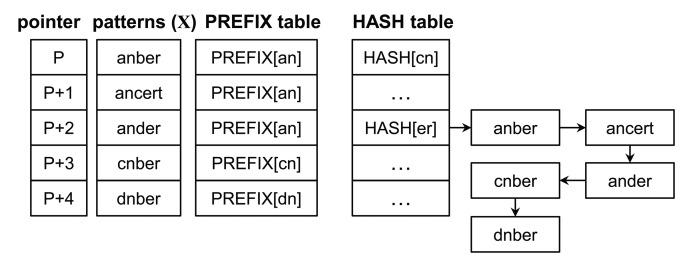
Sorted patterns and tables for the proposed algorithm.

Figure 5 shows the result of applying the prefix sorting operation. All patterns in the figure have the same suffix value and are sorted according to their prefix values as well as remaining characters. As a result, a pattern should have a prefix and character values greater than or equal to any preceding patterns in the list.

The prefix matching operation on *dnber* can be skipped according to the proposed Step 2. However, early termination by comparing a prefix value is less helpful when the number of patterns increases and there are many patterns within the same prefix value. For this reason, we also propose the early decision for character matching operations. This means that a character matching operation on *c* is only processed for *ancert*. Moreover, no character matching operation occurs for *ander*, and prefix matching operations for *ander*, *cnber* and *dnber* can also be skipped.

We have applied early decision to both the prefix and the character matching operations by including prefix sorting in the Wu–Manber algorithm. Moreover, it has been found that the overhead due to prefix sorting is negligible compared to the overall time taken to search.

### 3.3. Prefix Matching with Boundary Searching

In the previous subsection, we present two cases for the early termination of matching operations that efficiently reduce unnecessary prefix and character matching operations. However, there still exist the remaining prefix matching operations that can be further reduced. In this part, we present an extension of the early decision method based on boundary searching.

A pattern in the hash table entry is evaluated using the prefix matching operation followed by the character matching operation. Following this, subsequent patterns are also similarly evaluated until an early decision condition is satisfied. Patterns that have the same prefix and suffix are sequentially arranged after sorting. Therefore, if the boundary of the same prefix can be defined, matching operations for the same prefix patterns can be avoided. The following modifications are made to Steps 2 and 3:

Step 2: Compute the hash value *p* from the prefix characters in the current block. Using binary search, scan the prefix table entries for patterns listed in the *h* entry in the hash table to find the boundary of the same prefix group. If no such prefix exists, shift the block as in ASHIFT[*h*] and return to Step 1. Otherwise, go to the next step.
Step 3: For patterns within the boundary of the prefix group, the remaining characters of the pattern of which the prefix is matched with *p* are compared with the characters of the input text. The character matching operation continues until a mismatch occurs. When all characters in the pattern match those of the input text, the pattern is found. Return to Step 1. If all patterns in the boundary are evaluated or if a mismatch occurs and the character value of a pattern is greater than that of the input text, shift the block as in ASHIFT[*h*] and return to Step 1.

To eliminate unnecessary operations, the order of prefix matching operations is modified. In contrast with the previous algorithm, this new approach first filters out patterns with the same prefix and performs character matching operations only for the filtered patterns. To find the boundary of the prefix value over the sorted patterns, the binary search algorithm is used instead of sequential prefix matching.

## 4. Theoretical Analysis

This section presents a theoretical analysis of the overhead and efficiency of our proposed algorithm. We assume that all patterns are composed of randomly-generated and uniformly-distributed characters.

### 4.1. Average Number of Operations with Wu–Manber

The average complexity of the traditional Wu–Manber algorithm is known to be significantly affected by the length of an input sequence, the minimum length of a pattern, the number of characters used, the size of a block and the number of patterns [30]. In this paper, our analysis will focus on the number of matching operations.

In our analysis, the average number of prefix matching (PM) operations and the average number of character matching (CM) operations considered in a given scan window are obtained from Equations (1) and (2), respectively. First, the average number of PM operations is calculated as the number of patterns per suffix under the assumption that the patterns are uniformly distributed:

(1) $$E\{PM\}=\frac{N}{\Sigma^{B}}.$$

The average number of CM operations is calculated by considering that CM for a pattern is terminated as soon as any mismatch of characters occurs. Each character matching operation for a pattern can proceed to the next character only if the current character is matched. Consequently, the probability that a character matching operation proceeds to the next character is 1/Σ*^i^* for the *i*–th character. Therefore, the average number of matching operations for CM can be represented as:

(2) $$E\{CM\}=\frac{N}{\Sigma^{B+B^{\prime }}}\overset{M-B^{\prime }}{\underset{i=0}{\text{∑}}}\frac{1}{\Sigma^{i}},$$

where *B′* is the number of characters in a prefix block and *M* is the average length of patterns.

From Equations (1) and (2), the complexities of the Wu–Manber algorithm for each matching operation are *O*(*N*/Σ*^B^*) and *O*(*N*/(Σ*^B^*^+^*^B^*′)). Overall, the complexity of the matching operations of the Wu–Manber algorithm is *O*(*N*/Σ*^B^*).

### 4.2. Overhead Due to Auxiliary Shifting

We analyze the computational overhead due to auxiliary shifting operations on the preprocessing and matching processes. As mentioned in Section 3.1, while constructing the shift table, SHIFT[*h*] is initialized as *m* − *B* + 1 and updated to *n*SHIFT[*h*], which indicates a newly-computed value based on the current substring when SHIFT[*h*] > *n*SHIFT[*h*]. At this time, if *n*SHIFT[*h*] = 0, ASHIFT[*h*] = SHIFT[*h*]; then, SHIFT[*h*] = *n*SHIFT[*h*]. Therefore, there is no computational overhead caused by calculating auxiliary shift values during preprocessing.

In the matching process, the entry value *h* must be computed before accessing ASHIFT[*h*]. This *h* has been already calculated to access SHIFT[*h*] in Step 1. Therefore, there is no computational overhead from calculating *h*. However, an additional memory reading operation is required to access ASHIFT[*h*]. The complexity of this operation is *O*(1). This means that the complexity of the matching operations of the Wu–Manber algorithm with AS is equivalent to that of the traditional algorithm, as *O*(*N*/Σ*^B^*).

Unfortunately, the auxiliary shifting approach requires more memory to save AS than the traditional method. Each of the entries with zero shift values involves an auxiliary shift value. In the shift table, there are *N* zero values at most, where *N* is the number of patterns, because the entries corresponding to the suffix of the patterns only have a zero shift value. Therefore, the memory overhead for AS is less than or equal to *N* integer values. Consequently, the size of the shift table with AS is Σ*^B^* + *N* at most.

### 4.3. Average Number of Operations with Early Decision

The proposed algorithm uses the results of prefix sorting and implements them using the quicksort algorithm [32] during preprocessing. As is widely known, the quicksort algorithm has a complexity of *O*(*n* log *n*). The prefix sorting method rearranges *N*/Σ*^B^* patterns in each entry of the hash table, where *N* is the total number of patterns. Consequently, all patterns are sorted with a complexity of *O*(*N* log (*N*/Σ*^B^*)).

We now analyze the average number of matching operations for the early decision method. The number of prefix matching operations for the early decision is represented as *PM_ED_*, and the number of character matching operations is denoted by *CM_ED_*. Moreover, *PM_BS_* denotes the number of prefix matching operations through boundary searching.

We have considered the probability of proceeding to the next prefix matching operation in Step 2. For each prefix value, the given prefix value performs the prefix matching operation with the preceding prefix values of the patterns in the hash table. Therefore, the *i*–th prefix value should have as many as (*N*/Σ*^B^*^+^*^B^′*)*i* prefix matching operations. Therefore, the reduced average number of prefix matching operations can be represented as:

(3) $$E\{PM_{ED}\}=\frac{1}{\Sigma^{B^{\prime }}}\sum_{i=1}^{\Sigma^{B^{\prime }}}\frac{N}{\Sigma^{B+B^{\prime }}}i.$$

In Step 3, the early decision method skips the operations for the later patterns, which have larger character values. As a result, the method reduces the number of patterns that need to be evaluated by the character matching operation to *E*{*CM*}*i*/Σ, where *i* is the alphabetical order of a given character in the middle of the input text. Therefore, the average *CM_ED_* is represented as:

(4) $$E\{CM_{ED}\}=\frac{N}{\Sigma^{B+B^{\prime }}}\sum_{i=0}^{M-B^{\prime }}\frac{1}{\Sigma^{i}}\sum_{i=1}^{\Sigma }\frac{i}{\Sigma^{2}}.$$

From Equations (3) and (4), the complexity of *PM_ED_* is *O*(*N*/Σ*^B^*) and the complexity of *CM_ED_* is found to be *O*(*N*/(Σ*^B^*^+^*^B^′*)). As a result, the complexity of the overall matching operation becomes *O*(*N*/Σ*^B^*). In fact, the complexities of the early decision on the prefix and the character matching operations are identical to those of the Wu–Manber algorithm. Nevertheless, the method significantly reduces the expected number of matching operations. This is evaluated in Section 4.4.

Furthermore, we have shown the boundary searching prefix matching operation in Section 3.3. By changing the order of the matching operations, the prefix matching operation has been altered to two binary searches. Therefore, the average *PM_BS_* can be represented as:

(5) $$E\{PM_{BS}\}=2\log_{2}\frac{N}{\Sigma^{B}}.$$

With the method, the complexity of *PM_BS_* is *O*(log (*N*/Σ*^B^*)). Therefore, the overall complexity of the matching operations is reduced to *O*(*N*/(Σ*^B^*^+^*^B′^*)).

### 4.4. Performance Gain in Early Decision

We present the performance gain of our proposed method based on theoretical analysis in this subsection. In Section 4.3, we showed that *PM_ED_* and *CM_ED_* have fewer operations and the same complexity compared to the Wu–Manber algorithm. In contrast, *PM_BS_* has fewer matching operations, as well as lower complexity in the matching operations procedure compared to the Wu–Manber algorithm.

**Figure 6.**
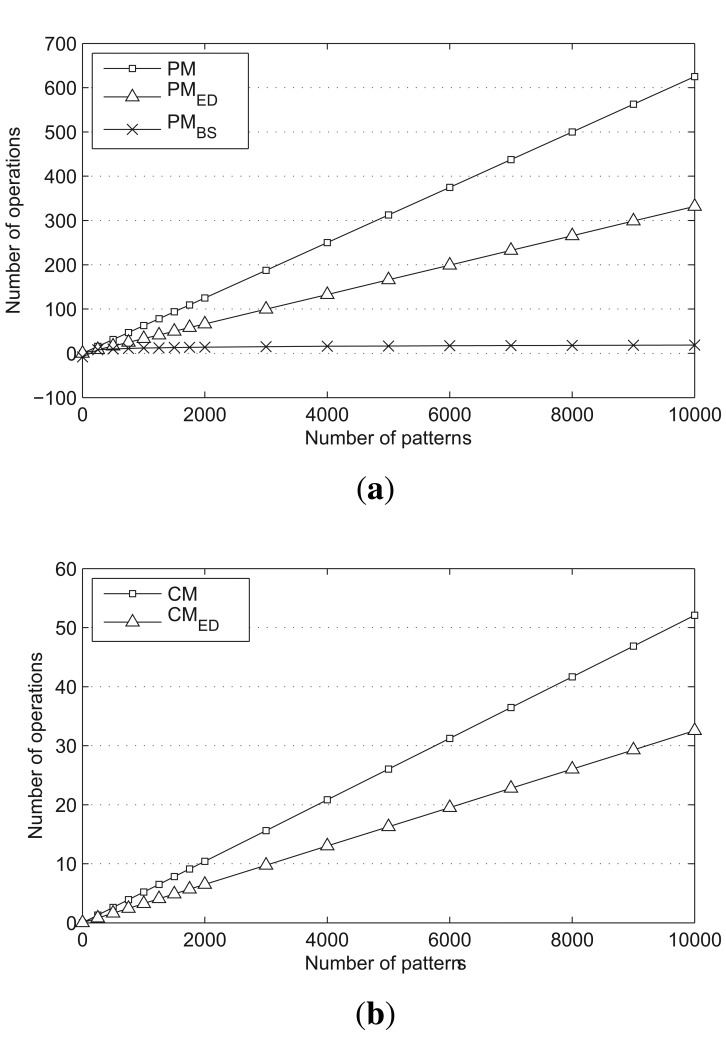
Number of matching operations based on theoretical analysis. (**a**) Prefix matching operation; (**b**) character matching operation.

The average number of operations for *PM, PM_ED_* and *PM_BS_* are compared in Figure 6a, and the average number of operations for *CM* and for *CM_ED_* are presented in Figure 6b. The size of the scan window and the prefix block are both set to two, and the number of characters is set to four. *PM_ED_* and *CM_ED_* reduce the number of required operations by nearly half. Moreover, *PM_BS_* shows very stable results, even when the number of patterns increases.

*PM_BS_* involves the smallest number of operations overall. However, there is a lower bound on the number of patterns required for *PM_BS_* to have fewer operations than *PM_ED_*. From the analysis in Section 4, we know that the average number of operations for Case 1 is approximately *N*/(2Σ*^B^*). Therefore, the number of patterns should be the larger value that satisfies:

(6) $$\frac{N}{2\Sigma^{B}}\geq 2\log_{2}\frac{N}{\Sigma^{B}}.$$

In other words, *PM_BS_* has fewer operations than *PM_ED_* when the number of patterns is greater than 16 · Σ*^B^*.

## 5. Implementation and Evaluation

In this section, we evaluate our proposed pattern-matching algorithm as a malicious pattern-detection engine for two kinds of security applications. This engine is implemented on software and tested on a real embedded system.

### 5.1. Implementation

**Figure 7.**
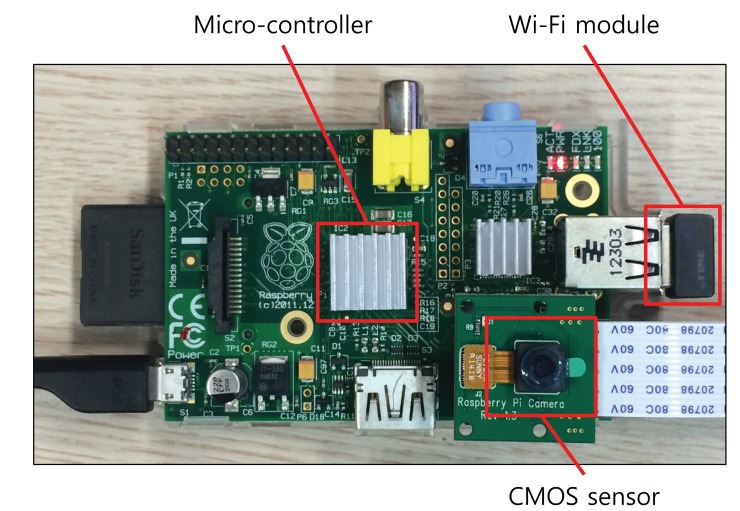
Secured IoT device architecture.

**Figure 8.**
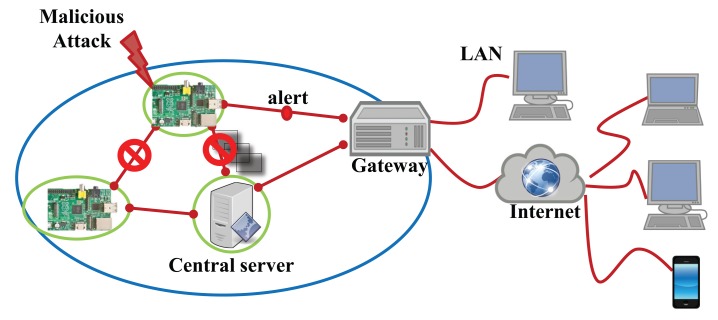
IoT network consisting of smart physical objects.

To evaluate the proposed security system for the IoT devices, we exploit a Raspberry Pi [33] computing unit integrating the Omnivision 5647 sensor as the IoT device shown in Figure 7. Many of the IoT systems use Raspberry Pi as the smart devices. The micro-controller of this device consists of an ARM1176 700 MHz processor, 256 MB of synchronous dynamic random-access memory (SDRAM) and 2 GB flash memory. Furthermore, this device is connected to the Internet using Wi-Fi. The main goal of this device is to capture 10 images of 640 × 480 pixels per second by the embedded sensor in a fixed focus module and to transmit these images to the central server. Our malicious detection system concurrently runs on the device during processing its main work streaming the captured images. If the system detects a malicious attack, it stops processing and communicating with the server and alerts the gateway, as shown in Figure 8.

The performance evaluation is carried out by using the traditional Wu–Manber algorithm (WM) and three proposed algorithms: auxiliary skipping (AS), early decision with boundary searching (EBS), and the approach that uses both AS and EBS (AS-EBS). EBS is composed of *PM_BS_* and *CM_ED_*. Furthermore, three previously proposed improvements to the Wu–Manber algorithm have been tested for comparison: Dai's quasi-multiple medium (QMM) algorithm [20], Hong *et al.*'s quick search improved WM algorithm (QWM) [27] and Lin *et al.*'s bakward hashing (BH) algorithm [28]. The block sizes (B) of the above algorithms are set to two, because of the limited memory of the embedded system. All algorithms are implemented in C, compiled using the O3 optimization level of the GNU Compiler Collection (GCC).

To test our proposed system, we use two kinds of intrusion pattern sets from Snort and ClamAV. Snort is a well-known open-source network IDS [34]. We exploit the Snort v2.9 rule set to obtain a pattern set containing 13,896 kinds of intrusions, including SMTP, DDoS, and DNS rules. In the case of ClamAV, which is an open-source anti-virus application [35], we have extracted 20,000 patterns from the non-polymorphic signatures of ClamAV. All patterns from Snort and ClamAV are sequences of characters from an alphabet Σ of size 256. The properties of these pattern sets are listed in Table 1. In addition, we exploit two types of input data streams. The target input data stream for Snort is 192 MB packet data captured from the Internet access. For ClamAV, we use target input data of 25 MB from the chain of binary files in the system directory.

### 5.2. Performance Results in Terms of Execution Time

**Figure 9.**
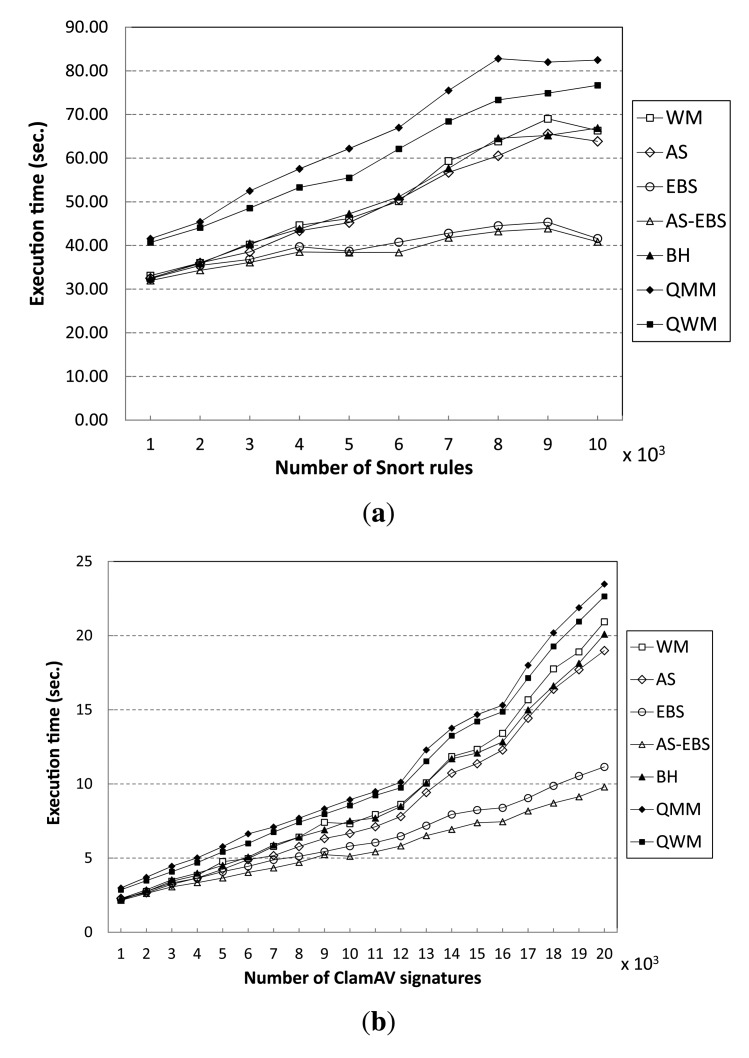
Performance results of various pattern-matching algorithms. (**a**) Snort; (**b**) ClamAV.

In order to test the performance of the proposed method, we show the execution times of all algorithms for matching varying numbers of patterns in Figure 9. From the results, we see that AS-EBS exhibits the best performance of all algorithms. For the Snort pattern set, AS-EBS attains a performance gain of approximately 62% over WM in matching 10,000 rules, as shown in Figure 9a. We believe this is because the auxiliary shifting approach enables one to shift by a greater number of characters in the input data stream than WM. Therefore, it can reduce the number of hashing operations following the completion of Step 2 of the matching procedure compared to WM.

Moreover, the early decision enables us to skip unnecessary matching operations in the prefix table (Step 2) and character matching (Step 3) in WM. AS attains a 4% performance improvement, and EBS records a speed-up of 1.59 compared with WM. For the ClamAV pattern set, AS-EBS again exhibits the best performance: it is almost 2.14-times faster than WM in matching 20,000 signatures, as shown in Figure 9b. AS records a 10% performance gain. Furthermore, EBS attains a speed-up of 1.87 compared to WM.

As the number of patterns increases, all algorithms suffer from performance degradation. However, the degradation in EBS and AS-EBS is much smaller than in the other algorithms tested. The execution time of EBS increases by a mere 28% to match 10,000 patterns instead of 1000 patterns in Snort. On the other hand, the execution time of WM doubles for the same increase in the number of patterns to match. The ClamAV pattern set shows similar results. The previous approaches, including BH, QMM and QWM, experience more significant performance degradation than EBS. We believe that these approaches require more memory for the additional table than WM in order to improve the average shift distance.

**Figure 10.**
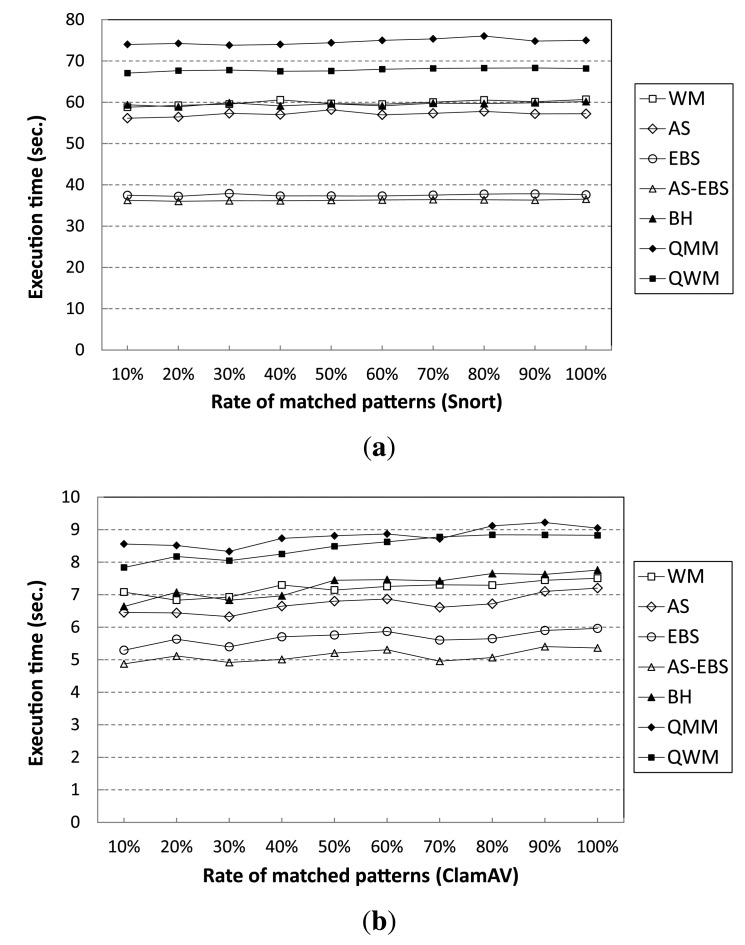
Performance results of different input data streams. (**a**) Snort; (**b**) ClamAV.

In addition, we have evaluated the performance when a large number of the Snort rules (patterns) appears in the target input data stream. Figure 10a shows execution time variation when different numbers of patterns exist in the input data stream. From the results, all of the algorithms show steady performance, although the percentage of the number of patterns that appears in the input data stream over 10,000 patterns increases from 10% to 100%. In fact, the performance gain of AS-EBS remains almost 60% compared to WM independent of the pattern matching rate. For the ClamAV pattern set, the execution time variation shows almost equivalent results to Snort; AS-EBS keeps the speedup factor of 1.4 compared with WM, regardless of the matching rate, as shown in Figure 10b.

### 5.3. Effect of Auxiliary Shift Value on Shifting Operations

**Figure 11.**
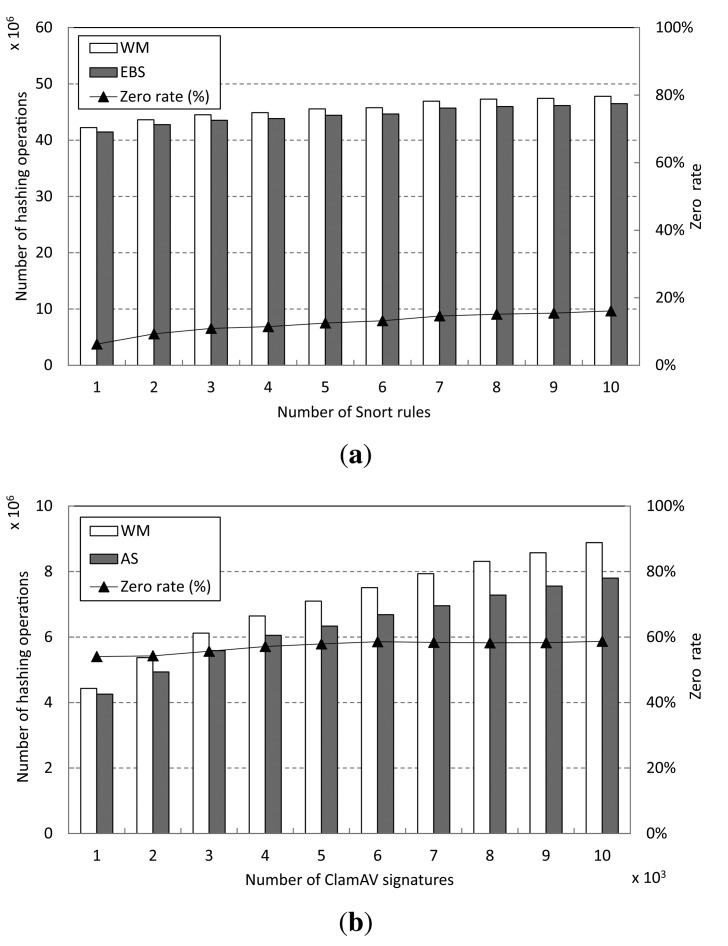
Number of hashing operations and zero rates for shifting operations. (**a**) Snort; (**b**) ClamAV.

The performance improvement achieved by the AS approach can be explained by the reduced number of hashing operations in Step 1 of the WM procedure. The zero rate, which is the number of blocks having zero shift values over the number of the shift table accessing operations, helps analyze performance improvement in detail. The zero rate is equal to the accessing rate of the auxiliary shift values. The number of hashing operations of WM and AS, as well as the zero rate, are depicted in Figure 11.

From the figure, we can see that AS has fewer hashing operations than WM. This is because of the larger number of characters that can be skipped in AS than in WM. As the zero rate increases, the number of skipped characters in AS grows. That is, the performance of AS improves in proportion to the zero rate. For the Snort pattern set, AS shows fewer hashing operations than WM, as shown in Figure 11a. On the other hand, a large number of hashing operations can be reduced by AS compared with WM for matching the ClamAV signatures, as shown in Figure 11b. This is because the zero rate for ClamAV is more than 50% and, thus, much higher than that for Snort.

### 5.4. Effect of Early Skipping on Matching Operations

**Figure 12.**
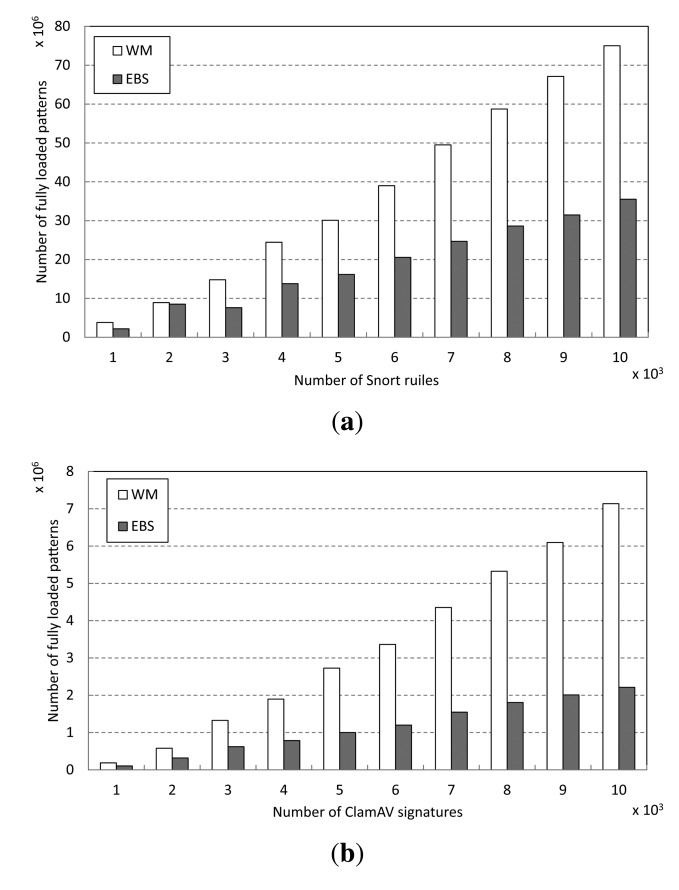
Number of fully-loaded patterns in the character matching step (Step 3). (**a**) Snort; (**b**) ClamAV.

To analyze the performance improvement achieved by early decision (EBS), we measure the number of patterns fully loaded in character matching operations in Step 3 of the matching procedure. The results are shown in Figure 12. For both of the pattern sets, Snort and ClamAV, EBS loads fewer patterns than the baseline WM in Step 3. For Snort, EBS reduces by more than half the number of fully-loaded patterns in matching over 7000 patterns, as shown in Figure 12a. Indeed, in the case of ClamAV, EBS loads only 30.7% of the number of patterns that WM does to match 10,000 signatures, as shown in Figure 12b. We believe this is because the proposed early decision scheme reduces the frequency in Step 3 due to the prefix matching operations and the computational complexity of the character matching operations in Step 3.

### 5.5. Performance Results in Terms of Preprocessing Time

**Figure 13.**
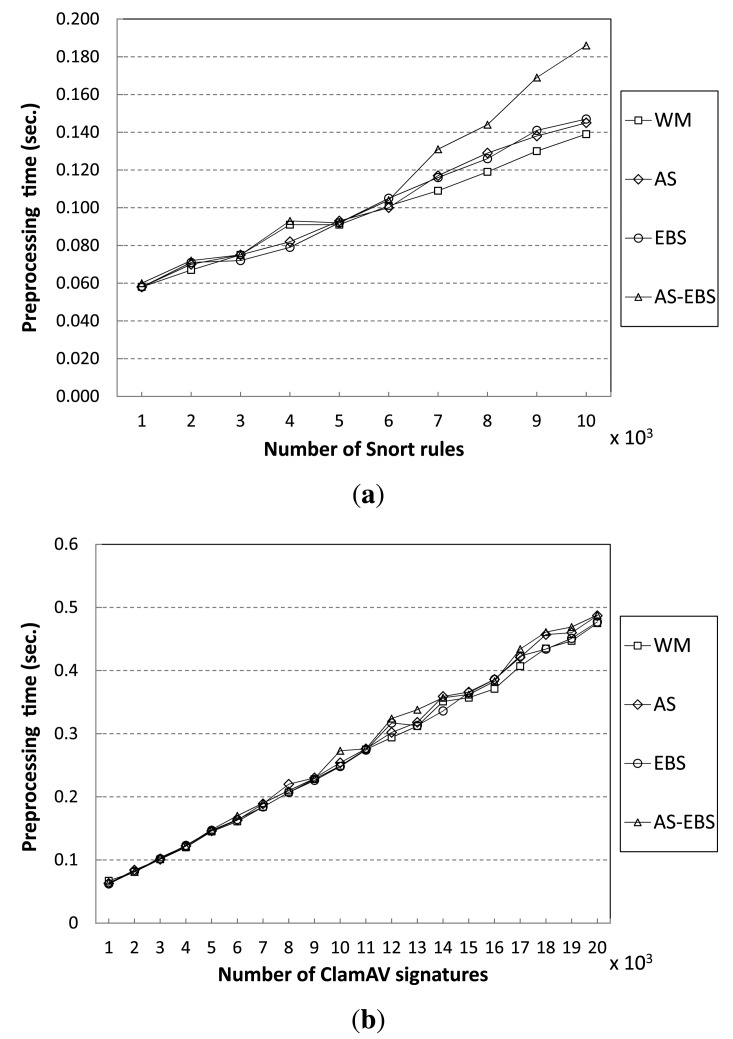
Preprocessing times of pattern-matching algorithms. (**a**) Snort; (**b**) ClamAV.

The proposed algorithms construct three required tables during the preprocessing stage before starting the detecting operations, as mentioned in Section 2.2. Figure 13 shows preprocessing times of the proposed algorithms and the WM algorithm for varying the number of patterns. In the Snort results, the proposed algorithms, including AS, EBS and AS-EBS, show similar preprocessing times compared to WM to match fewer than 6000 patterns. However, the overhead due to the AS and EBS approaches increases as the number of patterns grows to greater than 6000. AS requires 8% more preprocessing time than WM in the worst case to match 8000 patterns. This is because AS executes more memory allocating operations for the auxiliary shift values, the number of which is equal to the number of zero values in the shift table. Moreover, EBS also takes 8% more preprocessing time than WM to match 9000 patterns, because of pattern sorting. In the case of AS-EBS, there are overheads due to auxiliary shift values and pattern sorting. Therefore, it spends the longest on preprocessing; AS-EBS requires 33% more preprocessing time than WM.

For the ClamAV pattern set, the results are similar to that of Snort. Our approaches do not cause overhead during preprocessing to match fewer than 11,000 patterns. However, AS requires more time than WM to match more than 11,000 patterns. AS spends 5% more preprocessing time than WM to match 18,000 patterns. Furthermore, EBS requires 7% more time than WM. As a result, AS-EBS takes 10% longer than WM to preprocess data.

### 5.6. Detection Accuracy

**Figure 14.**
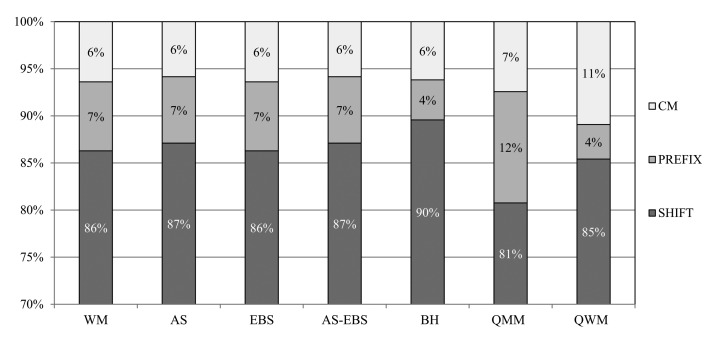
Detection accuracy of each matching steps.

In this subsection, the detection accuracies of the algorithms are evaluated. In fact, the low detection accuracy will cause a large number of matching operations. All of the algorithms based on WM consist of three matching steps: SHIFT, PREFIX and character matching (CM), as mentioned before. We measure the detection accuracies for each of these steps, respectively, and show the results for Snort in Figure 14.

From the results, AS and AS-EBS show 87% accuracy, which is 1% better than that of WM. This is because the auxiliary shifting scheme improves the detection accuracy for SHIFT by eliminating unnecessary shifting operations. However, the best accuracy for SHIFT is achieved by BH as 90%. This is 3% higher than that of AS. BH computes a shift value from twice as many as characters compared to the other methods. Therefore, BH requires more execution time than our approaches, although its detection accuracy for SHIFT is better than AS. Subsequently, the AS and AS-EBS approaches achieve 7% accuracy for PREFIX. This means that the detection accuracy after the prefix matching operation becomes 94%, which is the highest accuracy. Therefore, AS and AS-EBS perform the least number of character matching operations. Finally, all of the algorithms reach 100% accuracy after finishing CM. This is because CM performs exact character matching operations. In the case of EBS, the detection accuracy is equivalent to that of WM. In fact, the early skipping approach mainly targets eliminating unnecessary matching operations without the detection accuracy degradation from WM.

## 6. Conclusions

In this paper, we proposed a novel multiple pattern-matching algorithm for embedded security systems. Since the general embedded systems have a small size for the main memory, we limited the memory usage of the pattern-matching process. However, this limitation leads to performance degradation. To reduce the workload of the process, we proposed the auxiliary shifting method and the early decision scheme. The proposed methods successfully reduce the workload by skipping a large number of unnecessary matching operations through auxiliary shift values. The early decision of the matching operations, according to the prefix and character values, reduces the complexity of the prefix and character matching operations to a logarithmic scale.

Experiments showed that our proposed method achieved a speedup of up to 2.14 compared to the traditional pattern-matching algorithm given restricted resources. The proposed algorithm showed enhanced performance results especially when the number of patterns became large. Our proposed algorithm can thus contribute a high level of scalability to prevalent multiple pattern-matching algorithms.

## Figures and Tables

**Table 1.** Properties of pattern sets.

**Pattern Set**	**|Σ|**	**Number**	**Shortest**	**Avg.**	**Longest**
Snort	256	13,896	6	20	1021
ClamAV	256	20,000	16	204	694
